# The relationship between a low grain intake dietary pattern and impulsive behaviors in middle-aged Japanese people

**DOI:** 10.1371/journal.pone.0181057

**Published:** 2017-07-13

**Authors:** Atsuhito Toyomaki, Minori Koga, Emiko Okada, Yukiei Nakai, Akane Miyazaki, Akiko Tamakoshi, Yoshinobu Kiso, Ichiro Kusumi

**Affiliations:** 1 Department of Psychiatry, Graduate School of Medicine, Hokkaido University, Sapporo, Hokkaido, Japan; 2 Department of Public Health, Graduate School of Medicine, Hokkaido University, Sapporo, Hokkaido, Japan; 3 Center for innovation and Business Promotion, Hokkaido University, Sapporo, Hokkaido, Japan; Jadavpur University, INDIA

## Abstract

Several studies indicate that dietary habits are associated with mental health. We are interested in identifying not a specific single nutrient/food group but the population preferring specific food combinations that can be related to mental health. Very few studies have examined relationships between dietary patterns and multifaceted mental states using cluster analysis. The purpose of this study was to investigate population-level dietary patterns associated with mental state using cluster analysis. We focused on depressive state, sleep quality, subjective well-being, and impulsive behaviors using rating scales. Two hundred and seventy-nine Japanese middle-aged people participated in the present study. Dietary pattern was estimated using a brief self-administered diet-history questionnaire (the BDHQ). We conducted K-means cluster analysis using thirteen BDHQ food groups: milk, meat, fish, egg, pulses, potatoes, green and yellow vegetables, other vegetables, mushrooms, seaweed, sweets, fruits, and grain. We identified three clusters characterized as “vegetable and fruit dominant,” “grain dominant,” and “low grain tendency” subgroups. The vegetable and fruit dominant group showed increases in several aspects of subjective well-being demonstrated by the SF-8. Differences in mean subject characteristics across clusters were tested using ANOVA. The low frequency intake of grain group showed higher impulsive behavior, demonstrated by BIS-11 deliberation and sum scores. The present study demonstrated that traditional Japanese dietary patterns, such as eating rice, can help with beneficial changes in mental health.

## Introduction

Dietary habits affect mental health as well as physical well-being. For example, several studies have found that eating habits are associated with mental health, such as depression, sleep disturbances, severity of dementia, and subjective well-being.

Previous studies have defined and assessed dietary patterns in different ways. However, there is a claim that one should examine the association not of individual nutrition but of particular dietary patterns with psychological measures [1]. For example several previous studies assessed Japanese, Western, and Mediterranean dietary patterns and examined relationships with depressive state [2]. In addition, many other studies also reported dietary patterns associated with quality of sleep [3–6].

There are two analytic approaches for dietary patterns: the hypothesis-driven and the data-driven approach. The hypothesis-driven approach is an assessment method based on a priori defined combinations of food groups such as the Mediterranean diet. The data-driven approach is an exploratory method that uses multivariate statistical analyses to extract dietary patterns where large datasets demonstrating total food intake are aggregated and reduced to smaller datasets to summarize total dietary behavior [7]. Most studies exploring associations with mental health have focused on the data-driven approach. For example, recent studies using principal components analysis predicted depression [8, 9], dementia [10], sleep quality [4], attention deficit hyperactivity disorder [11], and subjective well-being [12].

Cluster analysis is the other data-driven analysis where dietary patterns are derived based on differences in mean dietary intake separating individuals into mutually exclusive, non-overlapping groups [13]. Cluster analysis has the advantage of producing dietary patterns that represent homogenous groups that can be associated with other variables [1]. In fact, several previous studies reported groups of individuals and their dietary patterns using cluster analysis [14–22].

For example, a recent Japanese study demonstrated that hemodialysis patients could be separated into three groups using cluster analysis and then the unbalanced patients group was identified to show adverse clinical outcomes [21].

As mentioned above, there are several methods to examine the relationship between dietary patterns and mental health. We are interested in identifying not a specific single nutrient/food group but populations preferring specific food combinations that can be related to some aspects of mental health. In addition, no study has examined the relationship between dietary pattern and multifaceted mental states using cluster analysis. The purpose of this study was to investigate population-level dietary patterns associated with mental state using cluster analysis. We focused on depressive state, sleep quality, subjective well-being, and impulsive behaviors using rating scales. We collected data from normal middle-aged people since they tend to have established dietary habits compared with younger people.

## Methods

### Participants

We asked 550 middle-aged Japanese people to respond to a self-administered questionnaire and then 351 subjects returned them. Of 351 subjects, we excluded 69 subjects with serious illnesses such as psychiatric disease, cancer, and metabolic syndrome. The remaining 282 participants consisted of 110 females and 172 males. Their ages ranged from 39 to 81 (M = 48.8, SD = 7.6). The local ethics committee from Hokkaido University approved this study. Written informed consent was obtained from all subjects.

### Dietary assessment (BDHQ)

Dietary pattern during the preceding month was estimated using a brief self-administered diet-history questionnaire (the BDHQ). The BDHQ is a 4-page structured questionnaire that calculates consumption frequency of selected foods to estimate the dietary intake of 58 food and beverage items [23]. Dietary intake can be estimated using a purpose-built computer algorithm based on the Standard Tables of Food Composition in Japan [24, 25]. The value of each item was energy-adjusted by density methods (g/1,000 kcal) in each subject.

### Depressive symptoms

The Japanese version of the Patient Health Questionnaire-9 (PHQ9) is a self-administered questionnaire that measures depressive symptoms [26]. Participants were asked to rate their experience of depressive symptoms in the past 2 weeks. Scores are calculated by assigning a score of 0, 1, 2, or 3 to the response categories of "not at all," "several days," "more than half the days," or "nearly every day," respectively. Higher scores indicate higher depressive symptomatology.

### SF-8

We used the Japanese version of the SF-8 as a health-related quality of life measure [27]. Each item of the SF-8 indicates a health profile of eight dimensions: General Health (GH), Physical Function (PF), Role Physical (RP), Bodily Pain (BP), Vitality (VT), Social Functioning (SF), Mental Health (MH), and Role Emotional (RE). In addition, the SF-8 produces two higher-order summary scores: the Physical Component Summary (PCS) and the Mental Component Summary (MCS). Both dimensions and the summary scores are standardized into values that range from 0 to 100 with a mean of 50 and a standard deviation of 10. Scores above and below 50 are considered above and below the average in the general Japanese population.

### Pittsburgh sleep quality index

We used the Japanese version of the Pittsburgh Sleep Quality Index (PSQI-J), which is a self-administered scale containing 9 items that inquire about frequency of sleep disturbances and subjective sleep quality during the previous month [28]. All item scores are summed to produce a global score ranging from 0–21.

### BIS-11

We used the Japanese version of the Barratt Impulsiveness Scale (BIS-11). It includes 30 items that inquire about impulsive behaviors. A recent study using secondary factor analysis for a Japanese sample indicated that the BIS-11 could be scored to yield four first-order factors (impulsiveness, planlessness, self-control, and deliberation) and two second-order factors (motor and non-planning impulsiveness). Therefore, we calculated four scores based on this finding.

### GPAQ

For participants' physical activity we used the Global Physical Activity Questionnaire (GPAQ). It assesses the following three aspects of physical activity by intensity (moderate or vigorous) as minutes per week: (1) occupational physical activity, (2) physical activity during transportation (walking, biking to work), and (3) recreational or leisure-time activity.

### Statistical analysis

Recently, K-means cluster analysis has been considered more desirable than conventional methods such as hierarchical cluster analysis. In fact, most previous studies using cluster analysis conducted K-means cluster analysis to identify groups that show discriminative dietary patterns [14, 16–20, 22]. Therefore, we performed K-means cluster analysis using thirteen BDHQ food groups: milk, meat, fish, egg, pulses, potatoes, green and yellow vegetables, other vegetables, mushroom, seaweeds, sweets, fruits, and grain. We excluded two food groups: drinking water and alcoholic drinks. Energy-adjusted food groups were converted to z-scores and entered into the cluster algorithm using SPSS (version 23). To determine the number of identified clusters, we analyzed three and four cluster solutions based on previous studies that used cluster analysis on a large Japanese sample using the DHQ (self-administered diet history questionnaire). The DHQ is a predecessor of the BDHQ and consists of a 16-page questionnaire that inquires about food consumption frequency and portion size to calculate the intake of 150 food and beverage items. Two studies indicated solutions of three clusters [17, 18] and one study indicated four clusters [19]. We performed K-means cluster analysis to test three-cluster and four-cluster solutions respectively. If a cluster contained <10 percent of the total sample, it was considered too small for adequate statistical power.

After the number of clusters was determined, values of each food group were adjusted by age and gender. Differences in mean rating scales about mental health across clusters were tested using ANOVA with Tukey-Kramer's adjustment for multiple comparisons, because the above-mentioned previous studies using cluster analysis performed ANOVA [14, 15, 17–20] or t-tests to examine group differences [16].

## Results

A total of 282 (110 female and 172 male) participants with complete data were included in this study. For the number of clusters, we decided to use a three cluster solution since it was reasonably sized (>10% of the sample size). The means and standard deviations of standardized value of each food consumption frequency across clusters demonstrated that the identified clusters varied in consumption frequency of key food groups. Cluster 1 (n = 72) was characterized by higher intake of fruit, vegetables, and milk. We labeled this the “vegetable and fruit dominant” group. Cluster 2 (n = 106) was characterized by higher intake of grain and thus was labeled the “grain dominant” group. Cluster 3 (n = 104) was characterized by extremely low intake of grain and thus was labeled the “low grain tendency” group.

**Fig 1:**
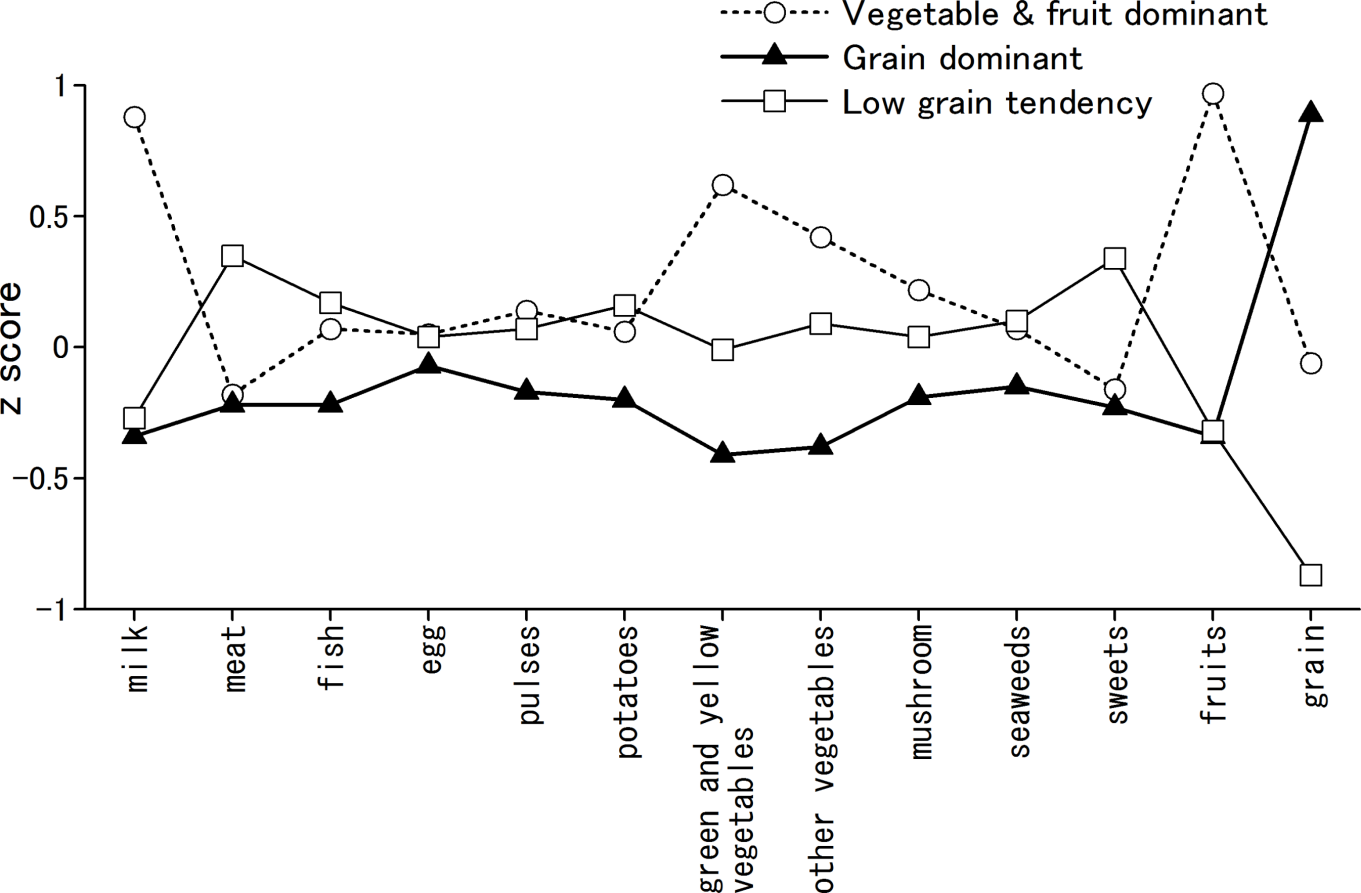
Profile of three dietary patterns identified by cluster analysis among 279 Japanese middle-aged healthy subjects.

Table 1 shows demographic information, standardized values of food groups, and rating scales. Regarding demographic information, a chi-squared test indicated significantly different sex ratios among the three groups. Regarding dietary patterns, ANOVA indicated significant main effects in several measures and subsequent analysis demonstrated significant differences among the three subgroups. Fig 1 shows the profiles of three dietary patterns identified by cluster analysis among 279 Japanese middle-aged healthy subjects. The “vegetable and fruit dominant” group showed extremely higher intake of milk, vegetables (green, yellow, and others), and fruits, and lower intake of meat. The “grain dominant” group showed extremely higher intake of grain (i.e., rice) and equal intake of other food groups. The “low grain tendency” group showed extremely lower intake of grain and higher intake of meat and sweets.

**Table 1:** Demographic information, standardized values of food groups, and rating scales about mental health.

	Vegetable & fruit	Grain dominant	Low grain	P*
N(female/male)	72 (37/35)	106 (29/77)	104 (44/60)	< .001
Age (SD)	50.50 (8.44)	48.23 (7.87)	48.33 (6.36)	0.97
BMI (SD)	22.72 (2.98)	22.64 (2.53)	22.76 (3.09)	0.97
Sitting time (minute) (SD)	388.42 (231.48)	406.73 (235.01)	426.25 (205.17)	0.54
Education (%)				0.59
<13 years	12.6	7.3	9.5	
13–14 years	25.2	23.6	19	
> = 15 years	62.2	69.1	71.4	
Occupation (%)	86.7	85.5	85.7	0.96
Cigarette smoking (%)				0.12
Never	50.4	52.7	33.7	
Former	29.5	29.1	41	
Current	20.1	18.2	25.3	
SF-8 GH (SD)	51.63 (6.49)	49.66 (5.76)	49.19 (5.92)	< .05
SF-8 PF	51.7 (4.29)	50.16 (5.89)	50.37 (4.44)	0.11
SF-8 RP	51.14 (5.03)	51.59 (3.94)	50.66 (4.83)	0.34
SF-8 BP	53.16 (8.15)	49.72 (7.77)	49.5 (8.4)	< .01
SF-8 VT	52.33 (5.9)	50.65 (5.57)	50.71 (5.86)	0.11
SF-8 SF	49.49 (7.56)	50.61 (6.43)	50.23 (6.53)	0.56
SF-8 MH	50.42 (6.36)	48.67 (6.36)	48.94 (5.89)	0.6
SF-8 RE	50.36 (5.25)	49.99 (5.22)	50.28 (5.02)	0.87
SF-8 PCS	51.35 (6.66)	49.77 (5.54)	49.18 (5.95)	0.06
SF-8 MCS	48.92 (6.96)	48.58 (5.81)	48.93 (5.98)	0.91
BIS-11 Impulsive (SD)	13.67 (3.3)	13.72 (3.35)	14.73 (3.08)	< .05
BIS-11 Planlessness	8.69 (2.41)	8.79 (2.04)	9.47 (2.23)	< .05
BIS-11 Self control	9.45 (2.56)	9.66 (2.46)	10.01 (1.96)	0.27
BIS-11 Deliberation	9.18 (2.18)	8.87 (1.87)	9.79 (1.73)	< .01
BIS-11 Sum	40.96 (7.45)	41.07 (7.05)	44.18 (6.05)	< .01
PHQ-9 (SD)	2.58 (3.27)	2.62 (3.21)	2.63 (2.6)	1
PSQI-J	4.4 (2.63)	4.02 (2.49)	4.05 (2.26)	0.54
GPAQ (minute) (SD)	1171.39 (1600.4)	1103.96 (1479.68)	1296.89 (1733.8)	0.68
milk(SD)	0.88 (1.07)	-0.34 (0.68)	-0.27 (0.85)	< .001
meat	-0.18 (0.87)	-0.22 (0.75)	0.35 (1.19)	< .001
fish	0.07 (1.09)	-0.22 (0.79)	0.17 (1.08)	< .05
egg	0.05 (1.01)	-0.07 (1)	0.04 (0.99)	0.63
pulses	0.14 (1.24)	-0.17 (0.78)	0.07 (0.99)	0.07
potatoes	0.06 (1.09)	-0.2 (0.77)	0.16 (1.11)	< .05
green and yellow vegetables	0.62 (1.22)	-0.41 (0.64)	-0.01 (0.9)	< .001
other vegetables	0.42 (1.24)	-0.38 (0.69)	0.09 (0.94)	< .001
mushroom	0.22 (1.25)	-0.19 (0.77)	0.04 (0.97)	< .05
seaweeds	0.07 (1.15)	-0.15 (0.7)	0.1 (1.13)	0.16
sweets	-0.16 (0.87)	-0.23 (0.68)	0.34 (1.23)	< .001
fruits	0.97 (1.19)	-0.34 (0.68)	-0.32 (0.6)	< .001
grain	-0.06 (0.7)	0.89 (0.69)	-0.87 (0.57)	< .001
drinking water	0.18 (1.22)	-0.07 (0.93)	-0.05 (0.88)	0.2
alcoholic drinks	-0.32 (0.64)	-0.24 (0.79)	0.47 (1.2)	< .001

* P value was calculated by ANOVA for continuous variables and the χ^2^ test for categorical variables to test differences between the clusters.

**Fig 2:**
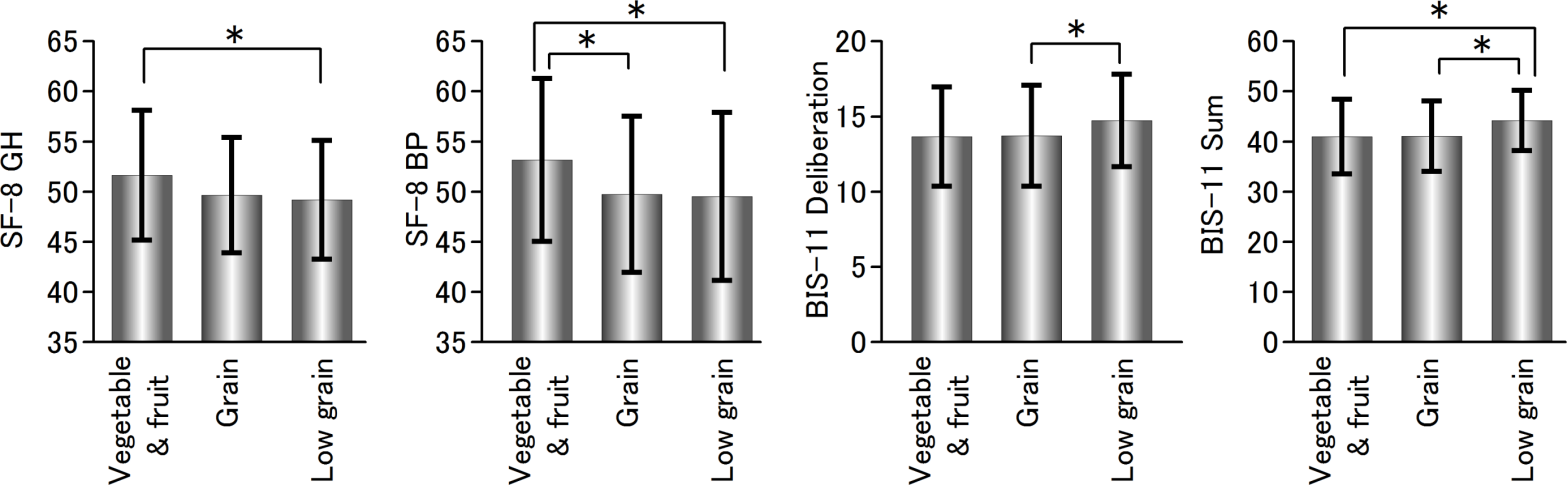
Rating scales about mental health where ANOVA and post hoc Tukey's test showed significant differences among three clusters.

Fig 2 indicates that rating scales about mental health where ANOVA and subsequent analysis showed significant differences among subgroups. Post hoc Tukey's test showed that the “vegetable and fruit dominant” group had significantly higher subjective well-being (SF-8 GH and BP) compared with the “low grain tendency” group (p < .05, respectively). On the other hand, a post hoc test showed that the “low grain tendency” group had significantly higher tendencies to impulsive behaviors (BIS-11 Deliberation and Sum) compared with the “grain dominant” group (p < .05, respectively).

## Discussion

The present study investigated the association of dietary patterns and various mental health measures among healthy middle-aged Japanese people using cluster analysis. We identified three clusters characterized as “vegetable and fruit dominant,” “grain dominant,” and “low grain tendency” subgroups. The vegetable and fruit dominant group showed increases in several aspects of subjective well-being demonstrated by SF-8 BP GH (General Health) and Bodily Pain (BP). The low frequency intake of grain group showed higher levels of impulsive behavior demonstrated by BIS-11 deliberation and sum scores.

The novelty of our finding was to reveal that low grain tendency, that is, a dietary pattern of low-frequency intake of rice and high-frequency intake of meat, was associated with higher levels of impulsive behaviors in middle-aged people. To our knowledge, no studies have been made on this point. For impulsive behaviors, one previous study investigated associations between dietary patterns in Korean school-aged children and attention deficit hyperactivity disorder (ADHD), which manifests in impulsive and inattentive behavior [11]. This study indicated that the traditional healthy dietary pattern in Korean food was associated with a lower odds ratio of ADHD. This study is similar to our study in showing the harmful effects of an anti-traditional dietary pattern. The low grain tendency pattern indicated in our study is incongruent with the traditional Japanese dietary pattern that emphasizes high-frequency intake of rice, fish, and soy beans foods.

Although more than twenty studies indicate significant associations of depressive state with specific dietary patterns, our study found no subgroup that showed a higher depressive tendency. When limited to studies of Japanese subjects, there are three studies that examined associations of dietary patterns with mood state including depressive state using the BDHQ. Suzuki and Miyaki [29] conducted a cross-sectional study of 2266 employees aged 21–65 years from all areas of Japan. Factor analysis identified three dietary patterns. Participants with a well-balanced Japanese dietary pattern showed significantly lower tendencies to mood/anxiety disorders. Nanri and Kimura [30] reported a cross-sectional study of 521 civil servants aged 21–67 years. Principal components analysis identified three dietary patterns: "Healthy Japanese," "Animal food," and "Westernized breakfast" dietary patterns. Participants with a healthy Japanese dietary pattern characterized by high intakes of vegetables, fruit, mushrooms, and soy products showed fewer depressive symptoms. On the other hand, Sugawara and Yasui-Furukori [31] conducted a study of 791 community-dwelling individuals aged 22–86 years. In particular, subjects were residents of a rural area of Hirosaki city, in northern Japan. Principal components analysis identified four dietary patterns: “Healthy,” “Western,” “Bread and confectionery,” and “Alcohol and accompanying” dietary patterns. No dietary patterns were associated with the risk of depression. Taken together, these results suggest that regional characteristics and working status affect associations of dietary patterns with mood state. Participants in our study varied in their working status and lived in the countryside or urban areas. In addition, our study differed from these previous studies by using cluster analysis. Statistical methods and characteristics of the participants may partially explain why we did not find dietary patterns associated with depression.

In summary, we have shown that a dietary pattern of low-frequency intake of rice and high-frequency intake of meat is associated with higher levels of impulsive behaviors in middle-aged Japanese people. Based on this finding, dietary advice that emphasizes a traditional Japanese dietary pattern can help in the improvement of public mental health.
